# Effects of semaglutide-loaded lipid nanocapsules on metabolic dysfunction-associated steatotic liver disease

**DOI:** 10.1007/s13346-024-01576-z

**Published:** 2024-04-13

**Authors:** Inês Domingues, Hafsa Yagoubi, Wunan Zhang, Valentina Marotti, Espoir K. Kambale, Katlijn Vints, Malgorzata Alicja Sliwinska, Isabelle A. Leclercq, Ana Beloqui

**Affiliations:** 1 UCLouvain, Université catholique de Louvain, Louvain Drug Research Institute, Advanced Drug Delivery and Biomaterials Group, Avenue Emmanuel Mounier 73, 1200 Brussels, Belgium; 2 EM-platform, VIB Bio Imaging Core, KU Leuven, Campus Gasthuisberg, Herestraat 49, 3000 Leuven, Belgium; 3 UCLouvain, Université catholique de Louvain, Institute of Experimental and Clinical Research, Laboratory of Hepato-Gastroenterology, Avenue Emmanuel Mounier 53, 1200 Brussels, Belgium; 4 WEL Research Institute, WELBIO Department, Avenue Pasteur, 6, 1300 Wavre, Belgium

**Keywords:** MASLD, MASH, Semaglutide, Rybelsus, GLP-1 analogs

## Abstract

Metabolic dysfunction-associated steatotic liver disease (MASLD) is a highly prevalent chronic liver disease that can progress to end-stage conditions with life-threatening complications, but no pharmacologic therapy has been approved. Drug delivery systems such as lipid nanocapsules (LNC) are very versatile platforms that are easy to produce and can induce the secretion of the native glucagon-like peptide 1 (GLP-1) when orally administered. GLP-1 analogs are currently being studied in clinical trials in the context of MASLD. Our nanosystem provides with increased levels of the native GLP-1 and increased plasmatic absorption of the encapsulated GLP-1 analog (semaglutide). Our goal was to use our strategy to demonstrate a better outcome and a greater impact on the metabolic syndrome associated with MASLD and on liver disease progression with our strategy compared with the oral marketed version of semaglutide, Rybelsus^®^. Therefore, we studied the effect of our nanocarriers on a dietary mouse model of MASLD, the Western diet model, during a daily chronic treatment of 4 weeks. Overall, the results showed a positive impact of semaglutide-loaded lipid nanocapsules towards the normalization of glucose homeostasis and insulin resistance. In the liver, there were no significant changes in lipid accumulation, but an improvement in markers related to inflammation was observed. Overall, our strategy had a positive trend on the metabolic syndrome and at reducing inflammation, mitigating the progression of the disease. Oral administration of the nanosystem was more efficient at preventing the progression of the disease to more severe states when compared to the administration of Rybelsus^®^, as a suspension.

**Graphical abstract:**

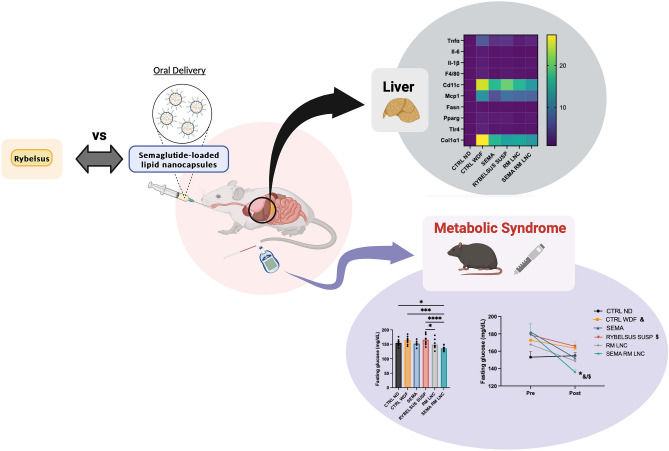

**Supplementary Information:**

The online version contains supplementary material available at 10.1007/s13346-024-01576-z.

## Introduction

Recently, a consensus on a new nomenclature has been proposed, with metabolic dysfunction-associated steatotic liver disease (MASLD) replacing nonalcoholic fatty liver disease (NAFLD) and metabolic dysfunction-associated steatohepatitis (MASH) replacing nonalcoholic steatohepatitis (NASH). This change was implemented due to problems with accurately capturing the etiology of the disease and in the use of stigmatizing language (“nonalcoholic” and “fatty”) [1]. In addition, the new definition includes at least one of five cardiometabolic risk factors, which are related to body mass index (BMI), fasting serum glucose, blood pressure, plasma triglycerides and plasma HDL-cholesterol levels, as diagnostic criteria [1, 2].

MASLD is a slow progressing chronic liver disease that results from a complex interplay of factors (environmental, genetic, lifestyle, etc.) [2]. These factors contribute to the gradual development of this highly prevalent metabolic liver disorder. The initial stage is known as metabolic dysfunction-associated steatotic liver (MASL), characterized by more than 5% of hepatocytes containing lipid droplets. Furthermore, MASH involves hepatic steatosis, lobular inflammation, hepatocyte injury with ballooning, and varying degrees of fibrosis. Advanced cases may lead to fibrosis, cirrhosis, and hepatocellular carcinoma (HCC) [2]. In a significant number of patients, MASLD coexists with other dysmetabolic traits, such as obesity and type 2 diabetes mellitus (T2DM), suggesting that MASLD is the hepatic manifestation of the metabolic syndrome (MetS), impacting approximately 30% of the global adult population [2, 3]. Existing treatment strategies primarily rely on lifestyle modifications, such as exercise and dietary restrictions, as the sole validated therapeutic intervention. Despite demonstrating efficacy in reversing MASH, maintaining life-style changes over the long term is challenging for most patients, and there are currently no pharmacological treatments approved [4].

Incretin-like hormones, such as glucagon-like peptide-1 (GLP-1), exert their effects by inducing glucose-dependent effects on insulin secretion, inhibiting glucagon release, slowing gastric emptying, and reducing food intake, ultimately leading to weight loss [5, 6]. The rapid inactivation of GLP-1 by the enzyme dipeptidyl peptidase IV (DPP-IV) within minutes prompted the development of GLP-1 analogs with prolonged half-lives, which are now approved for the treatment of T2DM and obesity (semaglutide, 160 h half-life). GLP-1 analogs indirectly impact hepatic metabolism via its direct effects on the pancreas and central nervous system. This actions leads to reduced hepatic steatosis, inflammation, and fibrosis, though to a lesser extent [6]. When administered via subcutaneous injection, GLP-1 analogs have been extensively investigated in clinical trials for MASLD treatment and show promise as mono- or combination therapies. Examples include liraglutide (LEAN Project: ClinicalTrials.gov, number NCT01237119) [7] and semaglutide (ClinicalTrials.gov, number NCT02970942) [8], both of which have completed phase II trials, meeting their primary endpoint of NASH resolution without worsening fibrosis. A phase III clinical trial for semaglutide has already been initiated (ClinicalTrials.gov, number NCT04822181). Moreover, Rybelsus^®^, an oral formulation of semaglutide, is currently being tested in 2 ongoing studies (ClinicalTrials.gov, numbers NCT05813249 and NCT03919929).

In previous research from our group, we demonstrated that oral drug delivery systems, such as lipid nanocapsules (LNC), effectively induce the release of endogenous GLP-1 [9]. Exenatide (EXE, half-life 2.5 h), an exogenous GLP-1 analog, has been successfully incorporated into these nanocarriers. This dual-action strategy (increased secretion of the native GLP-1 and increased absorption of a GLP-1 analog) has shown to be effective in a mouse model of T2DM. This approach not only impacted glucose homeostasis but also produced positive effects on hepatic steatosis, surpassing the outcomes observed with the subcutaneous injection of exenatide [9]. The oral administration of incretin mimetic peptides has the additional therapeutic advantage of simulating the normal physiological pathway of the native peptide. Orally administered GLP-1 analogs can access the liver at much higher concentrations via the hepatic portal vein than via subcutaneous delivery, reducing systemic exposure.

Therefore, we first selected exenatide to test the therapeutic effect of tour strategy on MASLD. We hypothesized that the increase in endogenous GLP-1 levels induced by lipid nanocapsules could reach therapeutic levels in the context of MASLD. In a previous MASLD study, we observed that exenatide-loaded lipid nanocapsules and blank nanoparticles (RM-LNC) had similar effects [10], which prompted the exploration of a more potent GLP-1 analog—semaglutide (SEMA). Therefore, we tested the effects of the blank and semaglutide-loaded lipid nanocapsules (SEMA-RM-LNC) on metabolic and liver parameters, in a rodent model of MASLD, and compared them with those of Rybelsus^®^, the oral marketed version of semaglutide that is available in the form of tablets and administered to the mice as a suspension.

The final goal of this study was to evaluate the impact of our nanosystem on the progression of the disease in a mouse model of early MASH (without fibrosis) when undergoing chronic treatment (one month) versus the oral administration of the peptide alone and its oral marketed version in a suspension form. Furthermore, we indirectly compared the results obtained with our previous studies using exenatide [10].

## Materials and methods

### Materials

Labrafac^®^ WL 1349 (caprylic/capric acid triglycerides) and Peceol^®^ (oleic acid mono-, di- and triglycerides) were obtained from Gattefossé (Saint-Priest, France). Lipoid^®^ S100 (soybean lecithin at 94% of phosphatidylcholines) was obtained from Lipoid GmbH (Ludwigshafen, Germany). Kolliphor^®^ HS15 (12-hydroxystearate PEG 660 and PEG 660), Span 80^®^ (Sorbitan Oleate) and sodium chloride (NaCl) were purchased from Sigma-Aldrich (St. Louis, USA). Semaglutide was purchased from Bachem (Bubendorf, Switzerland). Dipeptidyl peptidase IV (DPP-IV) inhibitor was purchased from Millipore (St. Charles, USA). Three mg Rybelsus^®^ tablets were purchased from a community pharmacy in Brussels. All chemical reagents used in this study were of analytical grade.

### Methods

#### Preparation of semaglutide-loaded lipid nanocapsules

LNC were prepared by a phase inversion temperature method using generally recognized as safe (GRAS) materials. Semaglutide was encapsulated within reserve micelles prior to its incorporation into LNC, as previously described by Xu et al. [9]. Briefly, semaglutide reverse micelles (RM) were prepared by mixing Labrafac^®^ WL 1349 and Span 80^®^ with high-speed stirring and then adding 50 μL of a 30 mg/mL solution of semaglutide in PBS with the pH adjusted to ~10–12 using 1 N sodium hydroxide (NaOH). LNC were prepared by weighing and mixing all the components (Labrafac^®^ WL 1349, Peceol^®^, Lipoid^®^ S100, Kolliphor^®^ HS15, sodium chloride (NaCl) and Milli-Q water). Then, this mixture was subjected to 3 temperature cycles of heating and cooling (50 °C–68 °C). During the last cycle, when the temperature was above the phase inversion zone (PIZ; 59 °C–61.5 °C), 500 μL of RM containing semaglutide was added to the mixture and allowed to cool down until the PIZ temperature was reached. Finally, 2.5 mL of cold Milli-Q water was added under stirring conditions. Blank LNC (RM-LNC) were prepared following the same protocol but without semaglutide. The composition of the nanocapsules is summarized in Table 1.

**Table 1:** Composition of the SEMA-RM-LNC

**LNC Composition**	
Lipoid^®^ S100 (mg)	13.4
Kolliphor^®^ HS15 (mg)	120
Peceol^®^ (mg)	85.5
Labrafac^®^ WL 1349 (mg)	769.5
NaCl (mg)	50
MilliQ Water (μL)	1025
Cold MilliQ Water (μL)	2500

**RM Composition**	
Labrafac^®^ WL 1349 (mg)	500
Span^®^ 80 (mg)	100
Semaglutide solution (30 mg/mL) in PBS or PBS (μL)	50

#### Characterization of semaglutide-loaded lipid nanocapsules

LNC were characterized in terms of their particle size, polydispersity index (PDI) and zeta potential. The first two parameters were assessed by dynamic light scattering (DLS), and the latter was assessed by laser Doppler velocimetry (LSV) using a Zetasizer Nano ZS (Malvern Instruments Ltd., Worcestershire, UK). For these analyses, 10 μL of LNC were dispersed in 2 mL of Milli-Q water. The encapsulation efficiency (EE, %) was calculated as follows: the total amount of semaglutide was calculated by disrupting the nanoparticles in methanol (50 μL of LNC in 950 μL of methanol) followed by strong vortexing. Free semaglutide was recovered by ultracentrifugation using Amicon^®^ centrifuge filters (MWCO 100 kDa, 4,000 g, 4 °C, 20 min) (Millipore, St. Charles, USA). The resulting filtrates were diluted 2 times. Total and free concentrations of semaglutide were quantified using a high-performance liquid chromatography (HPLC) method as described below. The drug loading (DL, %) was calculated as the total amount of semaglutide in the LNC, which was determined by HPLC, divided by the total amount of the SEMA-RM-LNC (the total amount of drug plus the total amount of LNC components). The EE (%) and LD (%) were calculated using the following equations:

$$EE\,\left(\%\right)=\frac{\left(Total \;amount \;of \;semaglutide\right)-(Free \;semaglutide)}{(Total \;amount \;of \;semaglutide)}\times 100$$

$$LD\, \left(\%\right)= \frac{(Total\; amount\; of\;semaglutide)}{(Total\; amount\; of\; SEMA\; RM\; LNC)} \times 100$$

#### Cryogenic transmission electron microscopy (cryo-TEM)

To further investigate the morphology and dimensions of the LNC, RM-LNC, and SEMA-RM-LNC specimens, a 10× dilution was prepared using Milli-Q filtered water. Subsequently, 3.5 μL of each sample was applied to a lacey grid that had been glow-discharged with a Leica ACE600 (Leica, Vienna, AT) coating unit within a humidity-controlled chamber of a Leica GP2 plunge-freezer. Following a 2-s back-blotting step, the grids were rapidly vitrified by plunging them into liquid ethane near its freezing point and then stored under liquid nitrogen. The samples were examined and imaged using a JEOL F200 (JEOL, Tokyo, JP) transmission electron microscope equipped with a Gatan Continuum energy filter and K3 camera. Zero loss filtering with a slit width of 20 eV was employed during imaging. The images were captured with a pixel size of 0.53 nm and a maximum exposure dose of less than 60 electrons per Angstrom [11]. Representative cryo-TEM images of RM-LNC and SEMA-RM-LNC can be found in Supplementary Information Fig. S1.

#### Quantification of semaglutide

The encapsulated concentrations of semaglutide were quantified by a high-performance liquid chromatography (HPLC, Shimadzu, Japan) gradient method as previously described by Xu et al. for exenatide [9]. A Kinetex^®^ EVO C18 column (100 Å, 2.6 μm, 150 × 4.6 mm) with a security guard column was used (Phenomenex, USA). The aqueous and organic mobile phases consisted of 0.05% (v/v) trifluoroacetic acid (TFA) in water and acetonitrile, respectively. The method uses a gradient of solvents with an initial ratio of 10:90 (v/v, aqueous: organic phase) at a flow rate of 1 mL/min linearly changing to 90:10 over 10 min and kept constant for one minute. After that, the ratio linearly changes to the initial composition during the next 1.5 min and stabilizes for one minute. The volume injected was 20 μL and the detection wavelength was 220 nm with a retention time of 7.5 min. The limits of detection and quantification were 1.8 ± 0.8 μg/mL and 5.3 ± 2.3 μg/mL, respectively.

#### In vivo studies

##### Animals

All animal studies were approved by and performed in accordance with the local animal committee under the reference 2023/UCL/MD/016.

##### Long-term treatment studies in an animal model of early MASH

Eight-week-old male C57BL/6 J mice were fed a normal diet (ND, SAFE Diets A03) or on a western diet containing 0.5% cholesterol (D05011404 Research Diets, USA) plus 30% (w/v) fructose (F0127, SigmaAldrich) in the drinking water for 20 weeks (WDF) (n = 10/group) [10]. The weight was monitored weekly. On the 18th week of disease induction and on the last week of treatment, the mice underwent a 4 h fasting period (the food was removed, and the fructose water was replaced with normal water), after which fasting glycemia was measured, and blood from the tail vein was collected (~60 μL) for insulin assessment. The treatment lasted for a period of 1 month while continuing the diet intake. Daily gavage administrations were given to the mice at the same time every day (3 pm), their weight was monitored, and non-fasting glycemia was measured every week. Semaglutide was administered orally in solution (SEMA) or encapsulated within reverse micelle lipid nanocapsules (SEMA-RM-LNC) (500 μg/kg). The commercial version of semaglutide in the form of oral tablets, Rybelsus^®^, was crushed for administration to mice, and a suspension was prepared for gavage at the same dose (500 μg/kg). The corresponding concentration of the unloaded lipid nanocapsules (RM-LNC) was also given orally. The control groups (CTRL ND/CTRL WDF) were given an equivalent volume of Milli-Q water by gavage. The volumes administered were based on the total amount of semaglutide present in the formulation using the HPLC method described above. After the treatment period, the mice were anesthetized with isoflurane (Isoflutek^®^, Karizoo Laboratories) and blood from the portal and cava veins was retrieved in the presence of a DPP-IV inhibitor (20 μL per mL of blood), after which the mice were euthanized by cervical dislocation. The blood collected was centrifuged (3,000 g, 15 min at 4 °C) and the plasma was stored at -80 °C for further analyses. Active GLP-1 was measured in portal plasma (ELISA kit, K1503OD Meso Scale Discovery, USA) whereas total GLP-1 (ELISA kit, K1503PD Meso Scale Discovery, USA) and liver enzymes (AST/ALT) (DRY-CHEM NX500, Fujifilm) were measured in systemic plasma. After the mice were sacrificed, their livers were collected and weighed. Liver sections were immersed in 4% paraformaldehyde (PFA) and embedded in paraffin for histological analysis. Another section was submerged in RNA stabilizing solution (RNA*later*^™^, Invitrogen, Thermo Fisher Scientific) and stored at 4 °C for 2 weeks for RNA preservation, after which the liquid was removed, and the samples were stored at -80 °C until further analysis. The remainder of the liver tissue was immediately snap frozen in lipid nitrogen and stored at -80 °C. The total lipid content was measured by extracting the lipids from frozen livers with methanol and chloroform and quantifying them by using the vanillin phosphoric acid reaction [12].

##### Histology and immunohistochemistry

Liver sections were stained with hematoxylin and eosin (H&E) or used for immunohistochemical detection of neutrophils. Briefly, a polyclonal rat anti-mouse LY-6G antibody (1:2000, BD Pharmingen 551459), a polyclonal rabbit anti-rat antibody (1:100, Vector AI-4001) and an envision anti-rabbit HRP (Dako K4003) were used, followed by a diaminobenzidine (Dako K3468)) to reveal the peroxidase activity, and then counterstained with hematoxylin. Neutrophils were quantified as the LY6G+ area (% total area) by using the QuPath software [13]. The NAFLD activity score (NAS) was assessed as previously described [14]. Briefly, NAS is defined as the sum of 3 histological features of NAFLD: steatosis ranging from 0 to 3 (0-<5%; 3->66%), ballooning ranging from 0 to 2 (0-none; 2-many) and finally lobular inflammation ranging from 0 to 3 (0-no foci; 3->4 foci). The score was blindly assessed, and the ballooning parameter was analyzed by IA.L., a hepatologist.

##### RNA extraction, reverse transcription and real-time qPCR

RNA was extracted from liver tissue using TRIzol (Invitrogen, Thermo Fisher Scientific, Belgium). Subsequently, 1 μg of RNA was utilized for cDNA synthesis, and gene expression was evaluated through quantitative polymerase chain reaction (Q-Rex, Qiagen, Hilden, Germany), following previously outlined procedures [15]. Ribosomal protein L19 (Rpl19) was used as a reference gene to normalize the mRNA levels. The genes analyzed are listed as follows: *Tnfα,* tumor necrosis factor alpha; *Il-6*, interleukin 6; *Il-1β*, interleukin 1 beta*; F4/80* (also known as Adgre1), egf-like module-containing, mucin-like, hormone receptor-like1; *Cd11c* (also known as Itgax), integrin alpha X; *Mcp1* (also known as Ccl2), monocyte chemotactic protein 1; *Fasn*, fatty acid synthase; *Pparg*, peroxisome proliferative activated receptor gamma; *Tlr4*, toll-like receptor 4 and *Col1a1*, collagen type 1, alpha 1. The sequences of primers used are listed in Supplementary Information Table S1.

##### Statistical analysis

The GraphPad Prism 10 (California, USA) program was used to perform all statistical analyses. The data are presented as the mean ± standard error of the mean (SEM) with outliers removed based on the Grubb’s test. Normality was assessed by using the Shapiro-Wilk test. For comparisons involving multiple groups a two-way or one-way ANOVA was performed followed by Tukey’s multiple comparisons test. A Kruskal-Wallis test was used, followed by Dunn’s multiple comparisons test for non-parametric analysis of multiple groups. For comparisons between 2 groups, an unpaired t-test or Mann-Whitney test was used to assess significant differences. A difference of *P* < 0.05 was considered to indicate statistical significance.

## Results and discussion

### Preparation and characterization of SEMA-RM-LNC

LNC with and without SEMA were prepared using generally recognized as safe (GRAS) excipients, and their composition is described in Table 1. The phase inversion method was performed as previously described [9]. The average size obtained was ~188.3 ± 3.40 nm, with a homogeneous population of nanoparticles (PDI < 0.2) and a negative surface charge (-9.6 ± 2.34 mV). The encapsulation efficiency of SEMA was 90.5 ± 0.82% (Table 2). Representative images of both RM-LNC and SEMA-RM-LNC are depicted in Supplementary Information Fig. S1.

**Table 2:** Physicochemical properties of semaglutide-loaded lipid nanocapsules (PDI: polydispersity index; EE: encapsulation efficiency (n=3))

**SEMA-RM-LNC**	**Size (nm)**	**PDI**	**Zeta Potential (mV)**	**EE (%)**
	184.8 ± 0.594	0.099	-9.29 ± 0.594	89.59
	188.4 ± 2.343	0.138	-12.14 ± 0.740	90.76
	191.6 ± 0.460	0.182	-7.50 ± 0.601	91.25
**Average**	**188.3 ± 3.402**	**0.140 ± 0.041**	**-9.64 ± 2.343**	**90.49 ± 0.815**

### SEMA-RM-LNC had a greater impact on the metabolic syndrome associated with MASLD than EXE-RM-LNC, RM-LNC and Rybelsus^®^

**Fig. 1:**
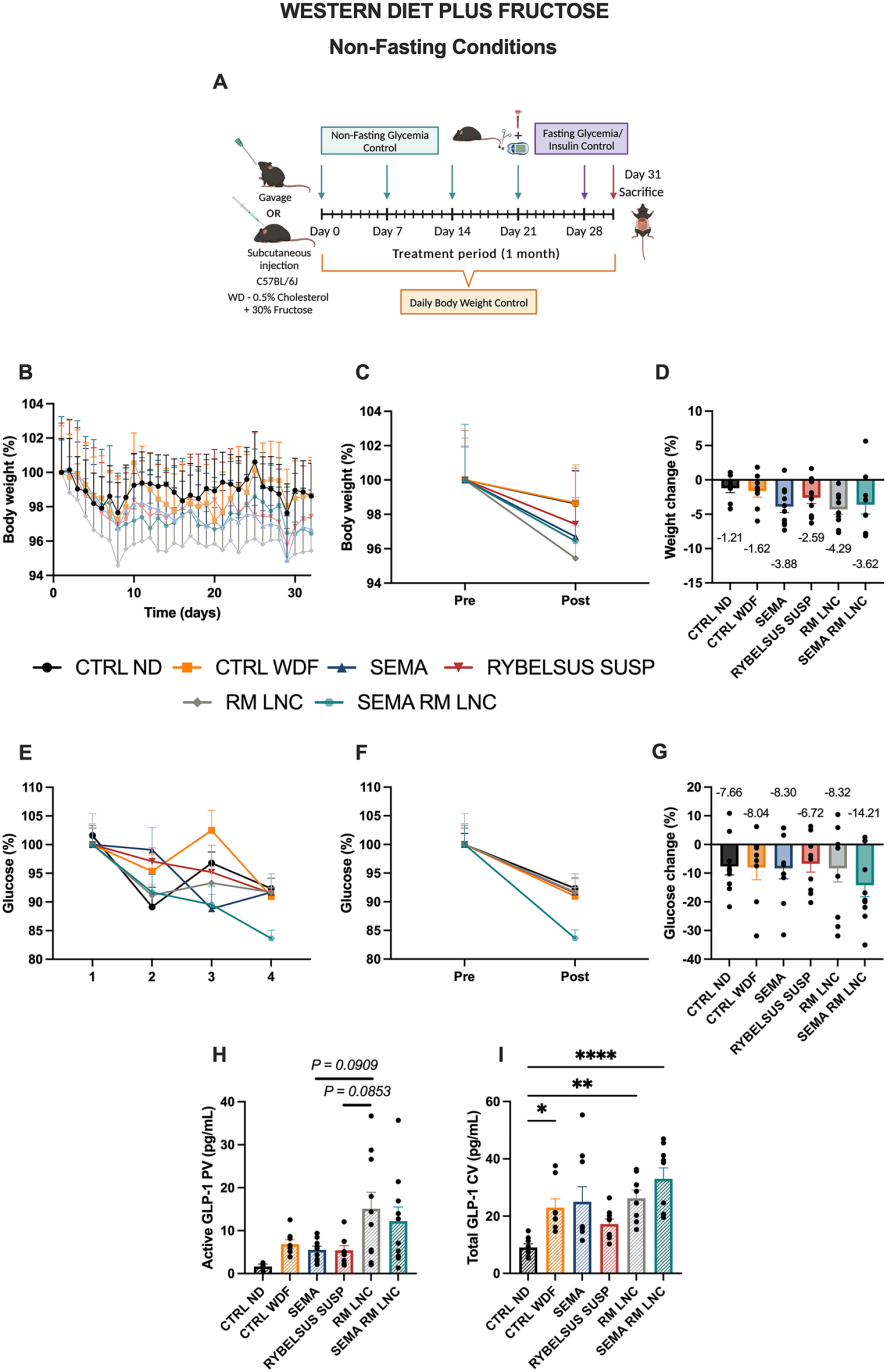
SEMA-RM-LNC has a greater impact on the metabolic syndrome than RM-LNC and Rybelsus^®^ under non-fasting conditions throughout the one-month treatment. **A** Schematic representation of the treatment period of 4 weeks, **B** Body weight (%), **C** Pre/Post: Body weight (%), **D** Body weight change (%), **E** Non-fasting glucose (%), **F** Pre/Post: Non-fasting glucose (%), **G** Non-fasting glucose change (%), **H** Active GLP-1 levels (pg/mL) measured in portal plasma, **I** Total GLP-1 levels (pg/mL) measured in cava plasma. Pre: beginning of treatment; Post: end of treatment. The results in **D**, **G** were calculated by subtracting the post values from the pre values. *P* values in **H**, **I** were determined by One-way Anova followed by Tukey’s post hoc test or the Kruskal-Wallis followed by Dunn’s post hoc test (*P < 0.05, **P < 0.01, ****P < 0.0001). The data are presented as the mean ± SEM (n = 9–10)

To assess the effectiveness of our treatment strategy in MASLD, an in vivo experiment in which C57BL/6 J mice were fed a western diet plus fructose in the drinking water (WDF) for 20 weeks was conducted. During the induction period of the disease, several parameters were analyzed: body weight gain, fasting glycemia, fasting insulin levels and HOMA-IR score. After 20 weeks of diet intake, the mice fed a WDF were obese with a weight gain of 24.60 ± 0.43 g compared to a 11.27 ± 0.52 g in the mice fed a normal rodent diet (ND).The average glycemia levels were 176.4 ± 3.86 mg/dL in the WDF group and approximately 153.2 ± 6.17 mg/dL in the ND group. Insulin levels were also significantly higher in the WDF-fed mice than in the ND-fed mice (ND: 1.060 ± 0.1 ng/mL vs. WDF: 2.027 ± 0.13 ng/mL). Similarly, the homeostatic model assessment of insulin resistance (HOMA-IR) was calculated, indicating the presence of insulin resistance, which is characteristic of MASLD and a major driver of the disease. The results are presented in the supplementary information (Fig. S2). The mice were then randomized into the different treatment groups to form body weight-matched groups (Supplementary information Fig. S3). After the 20 weeks, a 4-week daily chronic treatment was conducted, with daily gavage of either water, SEMA in solution, Rybelsus^®^ in suspension, RM-LNC (no SEMA) or SEMA-RM-LNC (500 μg/kg). In the clinic, semaglutide is given in 3 mg, 7 mg, or 14 mg doses. During this period, both fasting and non-fasting glycemia were assessed, and the body weight was controlled daily (Fig. 1A).

**Fig. 2:**
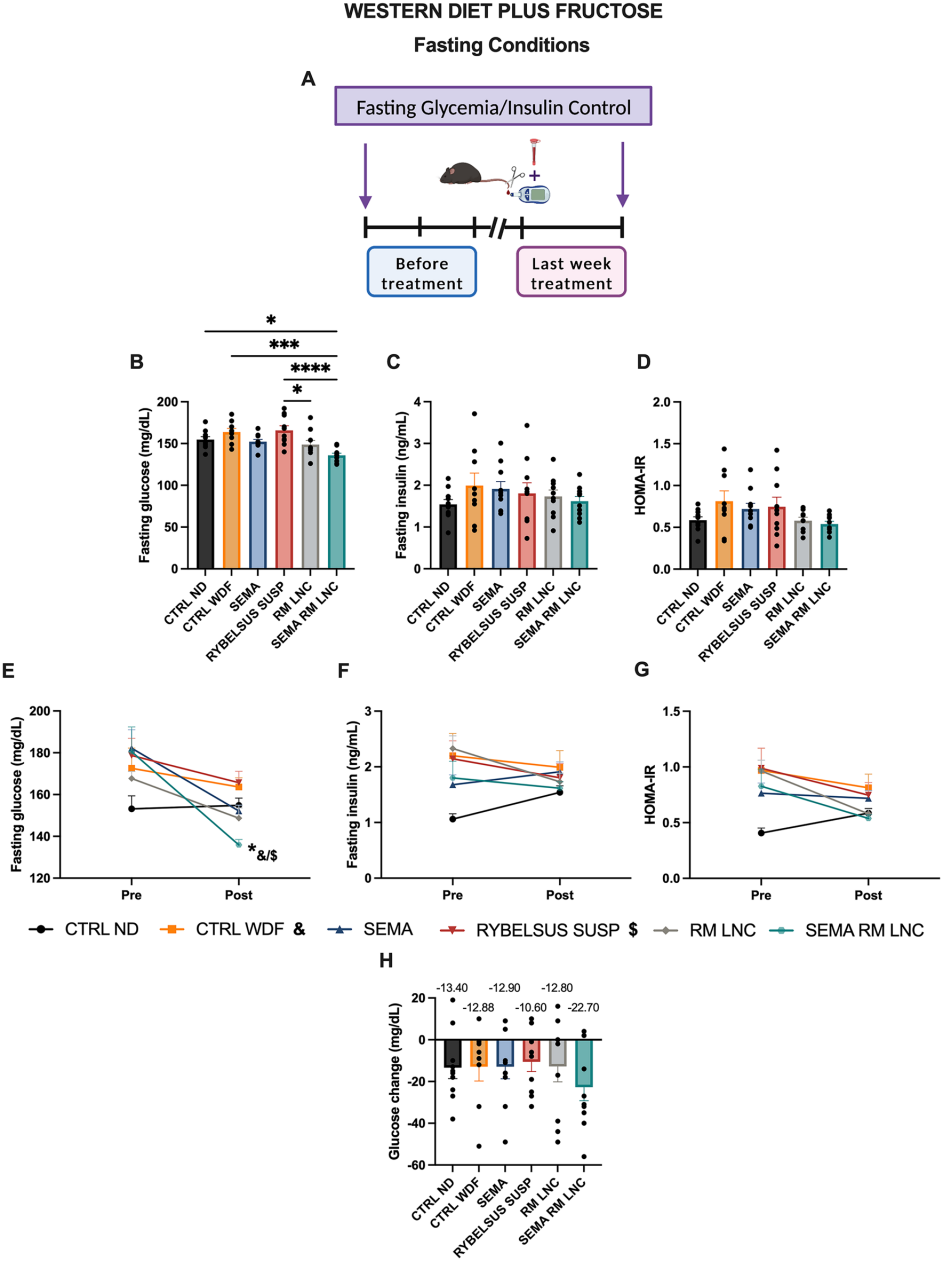
SEMA-RM-LNC has a greater impact on the metabolic syndrome than EXE-RM-LNC, RM-LNC and Rybelsus^®^ under fasting conditions throughout the one-month treatment **A** Schematic representation of the conduction of experiments under fasting conditions, **B** Fasting glucose (mg/dL), **C** Fasting insulin levels (ng/mL), **D** Homeostatic Model Assessment of Insulin Resistance (HOMA-IR) calculated using the equation [fasting glucose (mg/dL) x fasting insulin (ng/mL)/405], **E** Pre/Post: Fasting glucose (mg/dL), Pre/Post: Fasting insulin levels (ng/mL), **G** Pre/Post: HOMA-IR, **H** Fasting glucose change. The results in **H** were calculated by subtracting the post values from the pre values. *P* values in **B** were determined by One-way Anova followed by Tukey’s post hoc test. *P* values in **E** were determined by Two-way Anova followed by Tukey’s post hoc test (*P < 0.05, ***P < 0.001, ****P < 0.0001). The data are presented as the mean ± SEM (n = 9–10)

A 5 to 10% weight loss can lead to MASH resolution in humans [4] and in rodents [16]. In our study, all mice lost between 1 and 5% of their body weight, with no significant difference between the groups during the 4-week treatment period (Figs. 1 and S4), which is relatively short compared to the 48–72 week treatment period performed in clinical trials. Glycemia was measured under 2 different conditions: non-fasting glycemia was measured weekly, and fasting glycemia was measured before and at the end of the treatment period. Regarding non-fasting glycemia, although statistical significance was not observed, a positive trend was observed with SEMA-RM-LNC when compared to the other groups tested. Moreover, it should be highlighted that SEMA-RM-LNC had a better trend than SEMA ORAL, RYBELSUS SUSP and RM-LNC and better than our exenatide-loaded lipid nanocapsules (EXE-RM-LNC), which were used in our previous studies [10]. SEMA-RM-LNC decreased glycemia levels by approximately 14.2 ± 4.04% while glycemia levels in the other groups decreased by 6.7 to 8.3% (Figs. 1E–G and S4). In this study, we observed a better trend for glucose reduction with SEMA-RM-LNC than with the nanoparticles alone (Fig. 1E–G). We observed significant differences in the fasting glycemia values on the last week of treatment (POST), as mice treated with SEMA-RM-LNC exhibited a significantly lower fasting glycemia than almost all other groups, with almost normalized glycemia (Fig. 2B). The results from before and those at the end of the treatment period were compared and designated as PRE and POST, respectively. Significant differences in fasting glucose were detected between our strategy group (SEMA-RM-LNC) and the CTRL WDF and Rybelsus suspension group when comparing the PRE and POST results (SEMA-RM-LNC: 135.9 ± 2.59 mg/dL vs. CTRL WDF: 163.7 ± 4.36 mg/dL vs. Rybelsus: 165.7 ± 5.48 mg/dL) (Fig. 2E). Despite observing an effect on glycemia, we did not observe significant differences in the insulin (Fig. 2F) or HOMA-IR (Fig. 2G). This finding supports our hypothesis that a peptide with a long half-life might be more efficient in the context of MASLD treatment.

LNC can induce the secretion of the native GLP-1 when orally administered. This effect was previously demonstrated in a MASLD mouse model [10]. We further confirmed this by measuring GLP-1 levels in the portal blood 1 h after the gavage of fasted mice. We chose to measure it in the fasting state to avoid the interference with food intake, which can be modulated by the treatment itself. No significant differences in the levels of active GLP-1 were detected, but there was a trend toward higher levels of GLP-1 in the RM-LNC and SEMA-RM-LNC groups (Fig. 1H). In a previous study, we showed that empty LNC significantly increased the GLP-1 levels. The experimental conditions varied between the 2 studies, which may explain the difference. Indeed, here we measured levels 1 h after gavage, while in the previous study, we measured levels 30 min after treatment, and 30 min after an oral glucose load [10]. We did observe significant differences in total GLP-1 levels in systemic circulation, which were found to be higher in WDF animals than in control healthy animals but with no difference according to treatment. (Fig. 1I).

### SEMA-RM-LNC impact on liver steatosis and inflammation in early MASLD

**Fig. 3:**
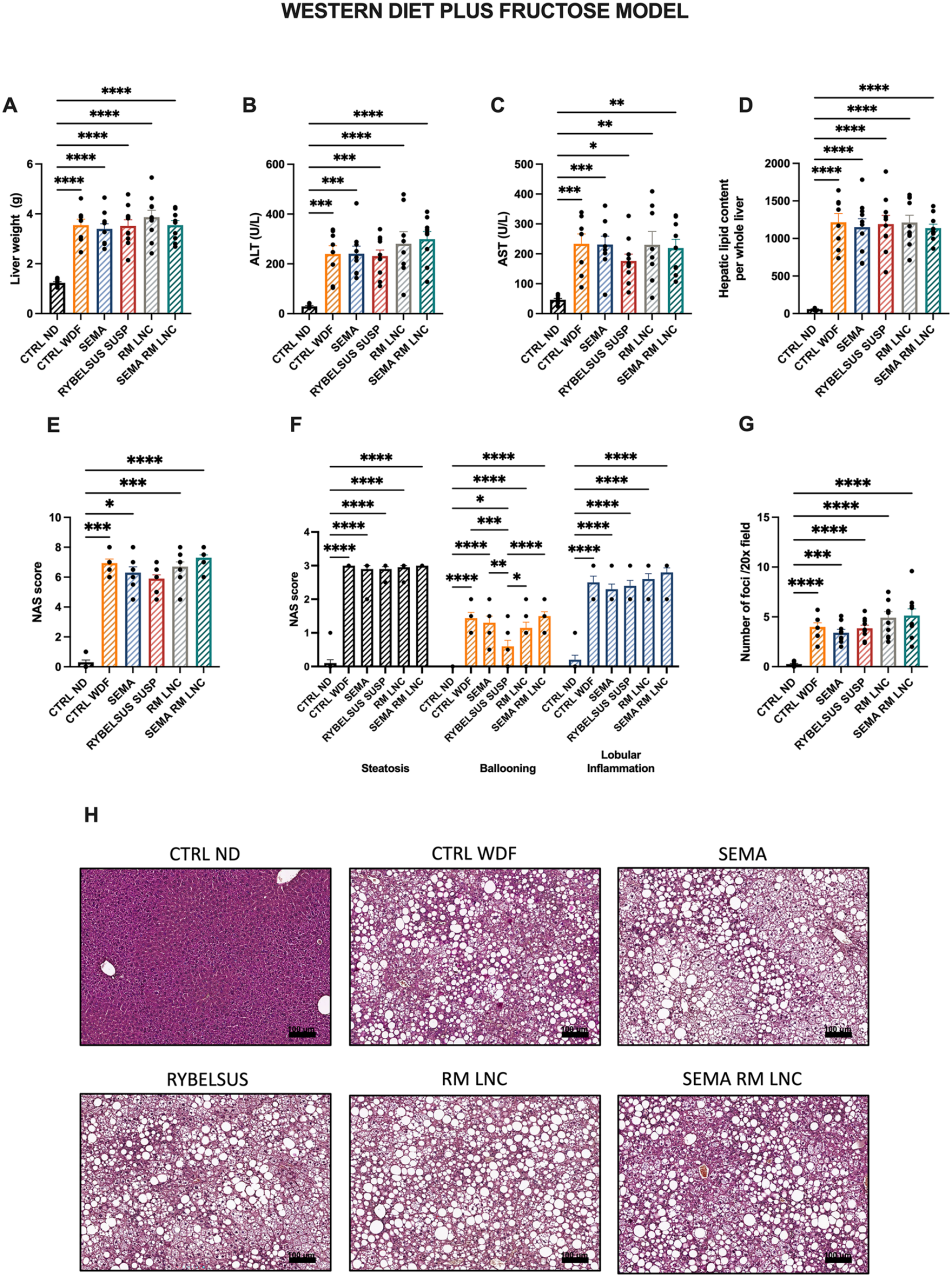
SEMA-RM-LNC has a similar effect on lipid homeostasis than EXE-RM-LNC, RM-LNC and Rybelsus^®^ **A** Liver weight (g), **B** ALT levels (U/L) measured in systemic plasma, **C** AST levels (U/L) measured in systemic plasma, **D** Total lipid content per whole liver, **E** Histological NAFLD activity score (NAS), **F** Steatosis, ballooning and lobular inflammation individual scores, **G** Inflammatory foci per 20× field, **H** Representative H&E liver sections (scale bar: 100 μm). *P* values were determined by One-way Anova followed by Tukey’s post hoc test or Kruskal-Wallis followed by Dunn’s post hoc test. *P* values in **F** were determined by Two-way Anova followed by Tukey’s post hoc test (*P < 0.05, **P < 0.01, ***P < 0.001, ****P < 0.0001). The data are presented as the mean ± SEM (n = 9–10)

Regarding the effect observed in the liver, we analyzed several markers relevant to the disease setting. We measured liver weight, liver transaminases (ALT and AST) and liver lipid content. Compared to those in the control group (CTRL ND), feeding mice WDF increased all these parameters, but we did not observe significative differences according to treatment (Fig. 3A–D). Aminotransaminase levels can be used as biomarkers for disease onset and consequent progression or amelioration because they indicate hepatic injury; however, their use in MASLD is not always specific. Alanine transaminase (ALT) tends to increase in MASLD and aspartate aminotransferase (AST) decreases; however, as the disease progresses to cirrhosis this ratio can reverse [17]. Several biomarkers are being investigated as possible and better indicators of disease progression with the aim of replacing the standard of diagnosis, which remains an invasive procedure (biopsy) [4, 18]. Histological liver slides were analyzed and scored for the presence of steatosis (% of tissue presenting steatosis), lobular inflammation (inflammatory cell infiltrates, foci, into the liver parenchyma) and ballooning (hepatocellular injury) [19]. In Fig. 3E, the NAS score is represented as the sum of these 3 features, with no significant differences between the groups fed a WDF. The NAS score was broken down and in the ballooning score, Rybelsus group was the only group with significant differences with all the other groups fed a WDF. Regarding the fat storage in the liver, histology (Fig. 3H), liver weight (Fig. 3A) and liver lipid content (Fig. 3D) confirmed that none of the treatments reduced steatosis, possibly due to the short duration (4 weeks) of treatment. Furthermore, in addition to a diet rich in fat and cholesterol, this model also contains 30% of fructose in the drinking water. Fructose, which is not metabolized via the same pathways as glucose, can increase de novo lipogenesis and further increase the fat storage in the liver [20, 21]. Several studies have shown the ability of GLP-1 analogs to reduce de novo lipogenesis, likely through indirect mechanisms [22]. However, this deserves further exploration.

**Fig. 4:**
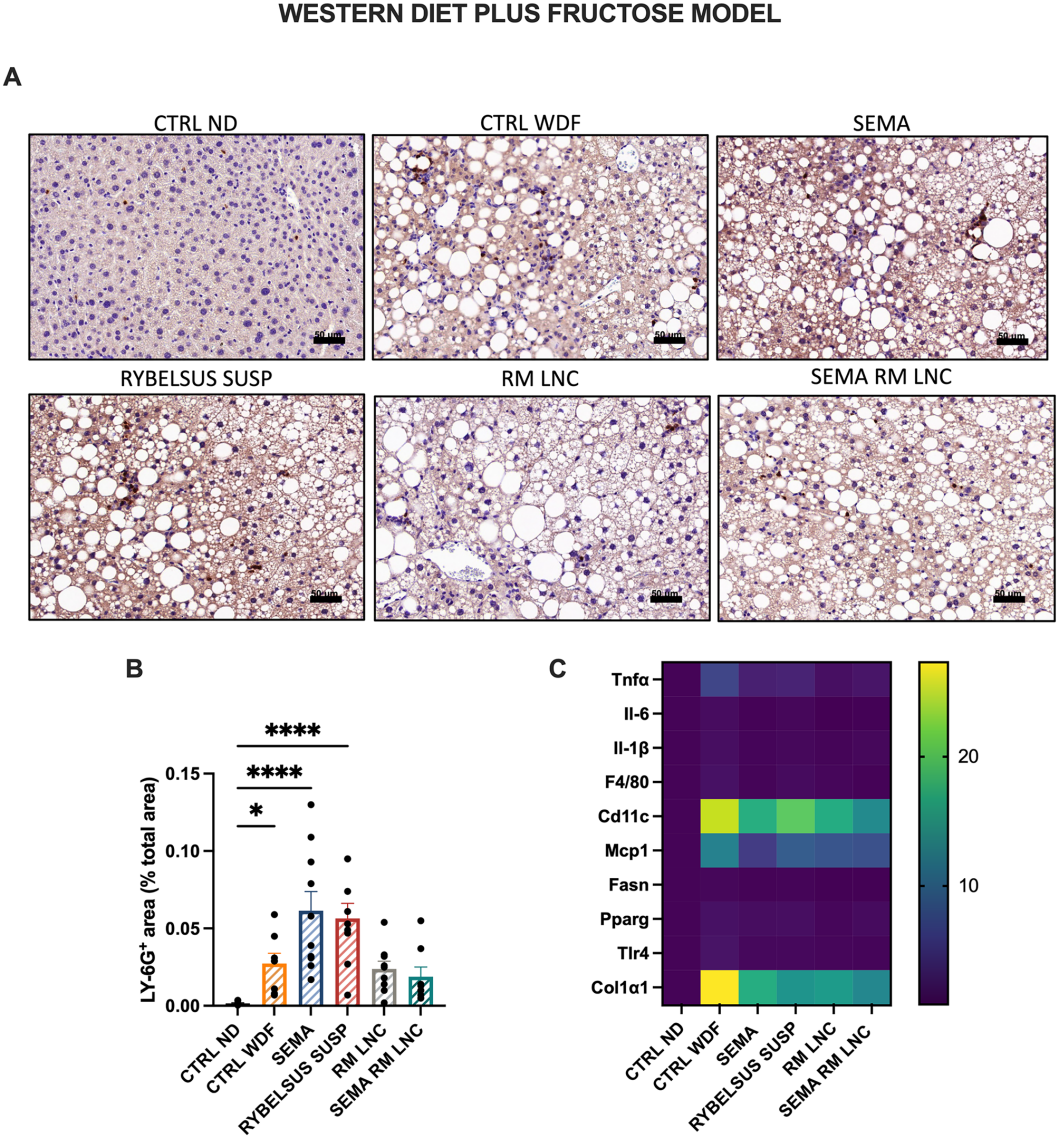
SEMA-RM-LNC reduces inflammation and infiltration/recruitment of immune cell populations in the liver **A** Representative LY-6G staining of liver sections per group (scale bar: 50 μm) **B** Quantification of neutrophils in liver sections, **C** Heatmap representation of the relative mRNA expression normalized to the CTRL ND group. P values were determined by One-way Anova followed by Tukey’s post hoc test or by Kruskal-Wallis followed by Dunn’s post hoc test (*P < 0.05, ****P < 0.0001). The data are presented as the mean ± SEM (n = 9–10)

In this study, we did not observe the effects of the treatments on the number of liver inflammatory cells (foci) (Fig. 3F, G). To determine the type of immune cell infiltrating the liver tissue, we conducted an immunohistochemistry assay to detect the presence of neutrophils. In chronic inflammatory diseases, such as MASLD, immune cells, including neutrophils, which are absent in the healthy tissue, are recruited to the liver. The role of these immune cells is not fully understood; however, they release toxic compounds, such as myeloperoxidase, which triggers additional production of reactive oxygen species, cytokines, and neutrophil extracellular traps (NETs), further aggravating the disease setting and progression [23]. In vivo studies revealed that mice lacking neutrophil elastase or myeloperoxidase had less liver damage. Furthermore, hyperglycemia seems to predispose neutrophils to produce more extracellular traps [24]. This ongoing cycle of damage contributes to further development of the disease, activating previously dormant hepatic stellate cells and initiating a fibrogenic state [23, 24]. The results showed that RM-LNC alone had a promising effect on neutrophil infiltration, as both RM-LNC and SEMA-RM-LNC were the only groups that were not significantly different from the CTRL ND group (RM-LNC: 0.024 ± 0.005% LY-6G^+^ area; SEMA-RM-LNC: 0.019 ± 0.006% LY-6G^+^ area; CTRL ND: 0.0016 ± 0.0003% LY-6G^+^ area) (Fig. 4A, B). Our nanoparticle group demonstrated superior enhancement in the management of metabolic syndrome, coupled with a more notable reduction in the levels of certain inflammatory markers, than the groups treated with semaglutide alone. Although further validation is needed, it is noteworthy that mice treated with semaglutide alone exhibited greater neutrophil infiltration. This can be attributed to the limited impact of semaglutide on both metabolic syndrome and the inflammatory state in the liver, unlike our treatment.

To further assess the impact of our treatment on disease progression we conducted qPCR assays analyzing key markers of immune cell infiltration, cytokine expression, endotoxin-mediated inflammation, lipid metabolism and fibrosis. Promising results were obtained at ameliorating inflammation with both RM-LNC and SEMA-RM-LNC (Figs. 4C and S5). Significant differences were detected between our SEMA-RM-LNC group and the diseased control group (CTRL WDF) in terms of the expression of markers related to cytokines (*Il-6*: *P = 0.0247), immune cell infiltration (*F4/80*: *P = 0.0334 and *Cd11c:* no significant differences with the CTRL ND), endotoxin-mediated inflammation (*Tlr4*: ****P < 0.0001) and fibrosis (*Col1α1*: *P = 0.0122) (Supplementary Information Fig. S4).

qPCR analysis revealed that the RM-LNC group exhibited results similar to those of the SEMA-RM-LNC group. Markers related to cytokine expression (*Il-6*: **P = 0.0062; *Il-1β*: **P = 0.0062), endotoxin-mediated inflammation (*Tlr4*: ***P = 0.0002), lipid metabolism (*Pparg*: **P = 0.0099) and fibrosis (*Col1α1*: *P = 0.0372) significantly differed between the RM-LNC group and the CTRL WDF group (Supplementary Information Fig. S5). Based on the literature, components present in lipid nanocapsules, such as phosphatidylcholine (PC), can have a therapeutic effect on the liver, mainly in hepatic steatosis [2, 25, 26]. PC is considered an essential phospholipid and its administration in preclinical and clinical studies has shown positive effects for reducing steatosis by increasing levels of polyunsaturated fatty acids and reducing the LDL/HDL ratio and TG levels [27–29]. In the context of MASLD, PC primarily contributes to enhancements in plasma lipid profiles, transaminase levels, and the inhibition of fat accumulation. Additionally, the use of PC has been associated with the downregulation of genes associated with pro-inflammatory macrophages, such as IL-6 [30]. The amount of LNC that reaches the liver after oral administration is currently unknown. However, we can hypothesize that if a significant amount of LNC ends up in the liver, and the components of the formulation do contribute to ameliorating the disease, further combined with their effect on GLP-1 secretion, this could explain the significant effect observed in liver inflammation and immune cell infiltration. Alternatively, as shown in Fig. 1H, the stimulation of GLP-1 release by the RM-LNC may alone explain this observation. Furthermore, the activation of the central nervous system by GLP-1 and GLP-1 analogs has been shown to reduce TLR-mediated inflammation which can explain the significant differences observed in the TLR4 marker analyzed by qPCR [31].

Overall, we observed a significant impact on glucose homeostasis with the SEMA-RM-LNC group (post-treatment) when compared to that of the other treatment groups. We did observe a greater impact on liver inflammatory markers such as *F4/80*, *Cd11c*, *Il-6*, *Tlr4* and *Col1α1* in the experimental group than in the CTRL WDF group. However, the magnitude of the effects was insufficient to significantly impact the liver weight, NAS score or liver histology. We can speculate that the treatment period is too short (4 weeks) and that prolonged treatment would be needed to yield a tangible positive effect, as reported by the 48 to 72 week long clinical trials [7, 8]. Moreover, the effect expected in the liver is thought to be indirect due to the lack of GLP-1 receptors in the liver. This possibly explains the non-significant effect of our treatment on some of the parameters analyzed and longer periods of treatment might be needed to start showing an effect. However, we believe that, considering the short period of administration, these results are promising towards the amelioration of the inflammatory state and glucose homeostasis. Further analysis can help us better understand the effect observed. For example, the evaluation of the inflammatory state present in the adipose tissue could be a good indicator of disease amelioration. Improving glucose homeostasis and reducing insulin resistance can initiate a decrease in the inflammatory state within adipose tissue. This, in turn, results in the release of fewer fatty acids into the bloodstream, mitigating their accumulation in the liver and subsequently reducing hepatic steatosis [2, 32]. Furthermore, determining whether the effect could be model specific, particularly considering the pro-lipogenic effect of fructose, which could mask a more subtle effect, will be important.

## Conclusion

Semaglutide was successfully encapsulated within LNC. A greater impact was observed in the metabolic syndrome associated with MASLD with the SEMA-RM-LNC than with the RM-LNC or Rybelsus^®^, in suspension form. Compared with the other treatments, SEMA-RM-LNC exerted a positive effect on glucose homeostasis when compared to the other treatment groups, in an animal model of early MASH. Moreover, our therapeutic approach showed promising effects on the inflammation observed in the liver, with a significant decrease in some of the markers analyzed. Furthermore, lipid nanocapsules alone also reduced liver inflammation and it is worth studying the mechanisms and/or the lipid composition responsible for this effect. To do so, we could tailor these nanostructures to have an optimal therapeutic effect both in the metabolic disorders and directly in the liver if a sufficient amount can reach the liver after oral administration. Longer treatment periods might be needed to demonstrate an effect, or combination therapy might be the way to go. This approach has potential for combination therapies via the oral route, potentially leading to novel approaches in MASLD treatment and oral incretin-based nanomedicine.

## Supplementary information

Below is the link to the electronic supplementary material.

Supplementary file1 (DOCX 2030 kb)

## Data Availability

Data will be available on request.
