# Supplementary material for: Effects of semaglutide-loaded lipid nanocapsules on metabolic dysfunction-associated steatotic liver disease
Source: Drug Deliv Transl Res. 2024 Apr 13;14(10):2917–29. doi: 10.1007/s13346-024-01576-z (PMC11385015; doi:10.1007/s13346-024-01576-z)
Supplement: Supplementary file 1 — Supplementary file1 (DOCX 2030 kb) [file 13346_2024_1576_MOESM1_ESM.docx]

**Semaglutide-loaded lipid nanocapsules effect on metabolic dysfunction-associated steatotic liver**

Inês Domingues^1^, Hafsa Yagoubi^1^, Wunan Zhang^1^, Valentina Marotti^1^, Espoir K Kambale^1^, Katlijn Vints^2^, Malgorzata Alicja Sliwinska^2^, Isabelle Leclercq^3*^, Ana Beloqui^1,4*^

^1^UCLouvain, Université catholique de Louvain, Louvain Drug Research Institute, Advanced Drug Delivery and Biomaterials Group, Avenue Emmanuel Mounier 73, 1200 Brussels, Belgium

^2^EM-platform, VIB Bio Imaging Core, KU Leuven, Campus Gasthuisberg, Herestraat 49, 3000 Leuven, Belgium

^3^*UCLouvain,* Université catholique de Louvain, Institute of Experimental and Clinical Research, Laboratory of Hepato-Gastroenterology, Avenue Emmanuel Mounier 53, 1200 Brussels, Belgium

^4^WEL Research Institute, WELBIO Department, Avenue Pasteur, 6, 1300 Wavre, Belgium

*Corresponding authors: [isabelle.leclercq@uclouvain.be](mailto:isabelle.leclercq@uclouvain.be) & [ana.beloqui@uclouvain.be](mailto:ana.beloqui@uclouvain.be)

**
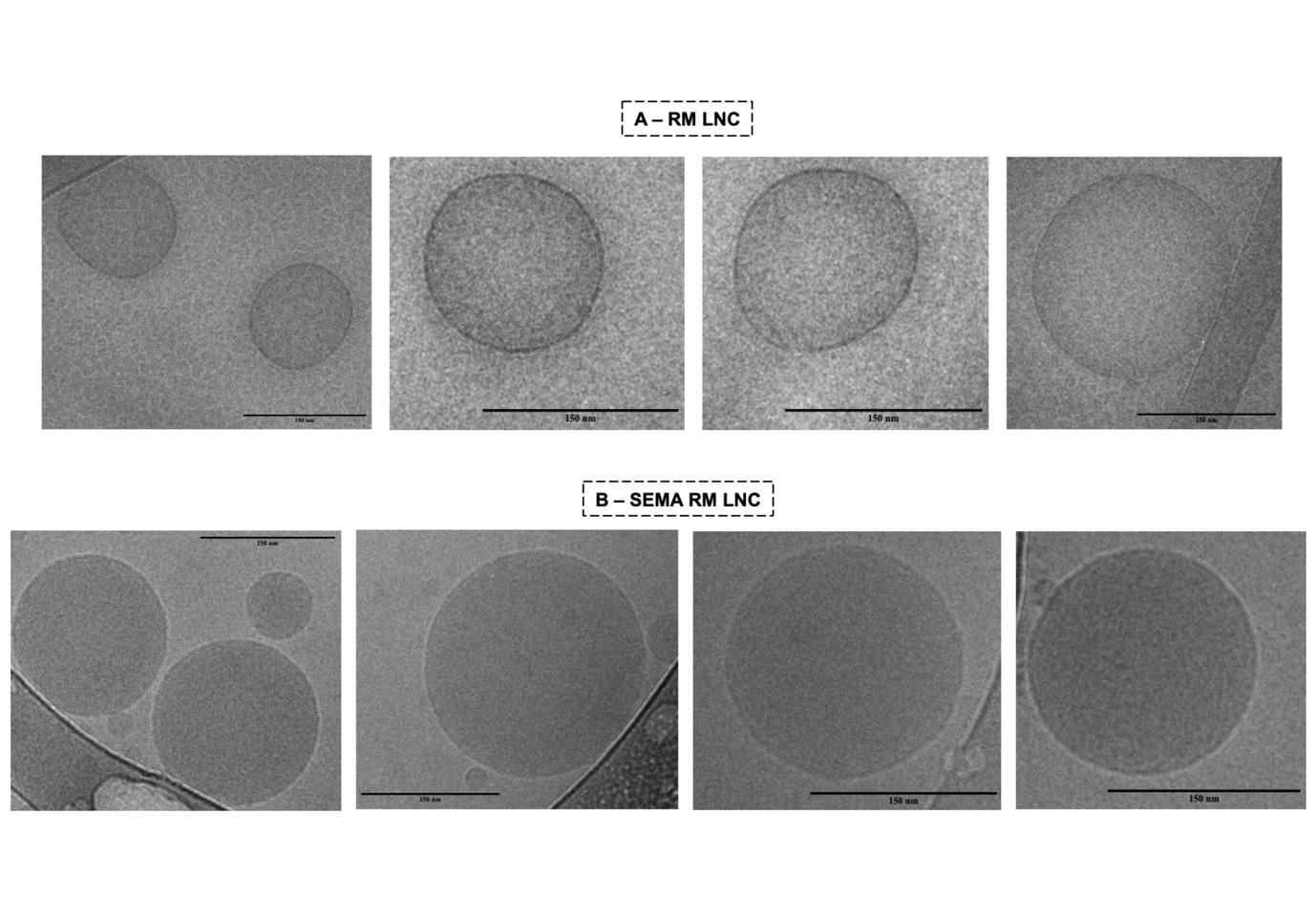
Supplementary Figures/Tables**

**Figure S1:** Cryo-TEM representative images of both RM LNC and SEMA RM LNC (scale bar: 150 nm)

**Figure S2:** Dietary model of NASH – Western diet plus fructose model: disease induction WDF vs ND (A) Schematic representation of the disease induction period before starting the treatment (20 weeks), (B) Body weight (g) throughout the 20 weeks, (C) Body weight gain (g), (D) Fasting glucose (mg/dL), (E) Fasting insulin (ng/mL), (F) Data shown in (D-F) were obtained at week 18 of disease induction. P values in (B) were determined by Two-Way ANOVA followed by Šídák's post hoc test. P values in (C-F) were determined by Unpaired t-test or Mann-Whitney test (**P*<0.05, ***P<*0.01, *****P<*0.0001). Data represented as mean±SEM (n=10-50).

**Figure S3:** Dietary model of NASH – Western diet plus fructose model: disease induction per groups (A) Schematic representation of the disease induction period before starting the treatment (20 weeks), (B) Body weight (g) throughout the 20 weeks, (C) Body weight gain (g), (D) Fasting glucose (mg/dL), (E) Fasting insulin (ng/mL), (F) Homeostatic Model Assessment of Insulin Resistance (HOMA-IR) calculated using the equation [fasting glucose (mg/dL) x fasting insulin (ng/mL)/405]. Data shown in (D-F) were obtained at week 18 of disease induction. P values in (B) were determined by Two-Way ANOVA followed by Tukey’s post hoc test. P values in (C; E; F) were determined by Kruskal-Wallis followed by Dunn’s post hoc test (**P*<0.05, ***P<*0.01, *****P<*0.0001). Data represented as mean±SEM (n=9-10).

**Figure S4:** SEMA-RM-LNC have an impact on glucose homeostasis and insulin resistance in the WDF model of early NASH. (A) Body weight (g), (B) Pre/Post: Body weight (g), (C) Body weight change (g), (D) Non-fasting glucose (mg/dL), (E) Pre/Post: Non-fasting glucose (mg/dL), (F) Non-fasting glucose change (mg/dL). Pre: beginning of treatment; Post: end of treatment. Results in (C; F) were calculated by subtracting the post values from the pre values. Data represented as mean±SEM (n=9-10).


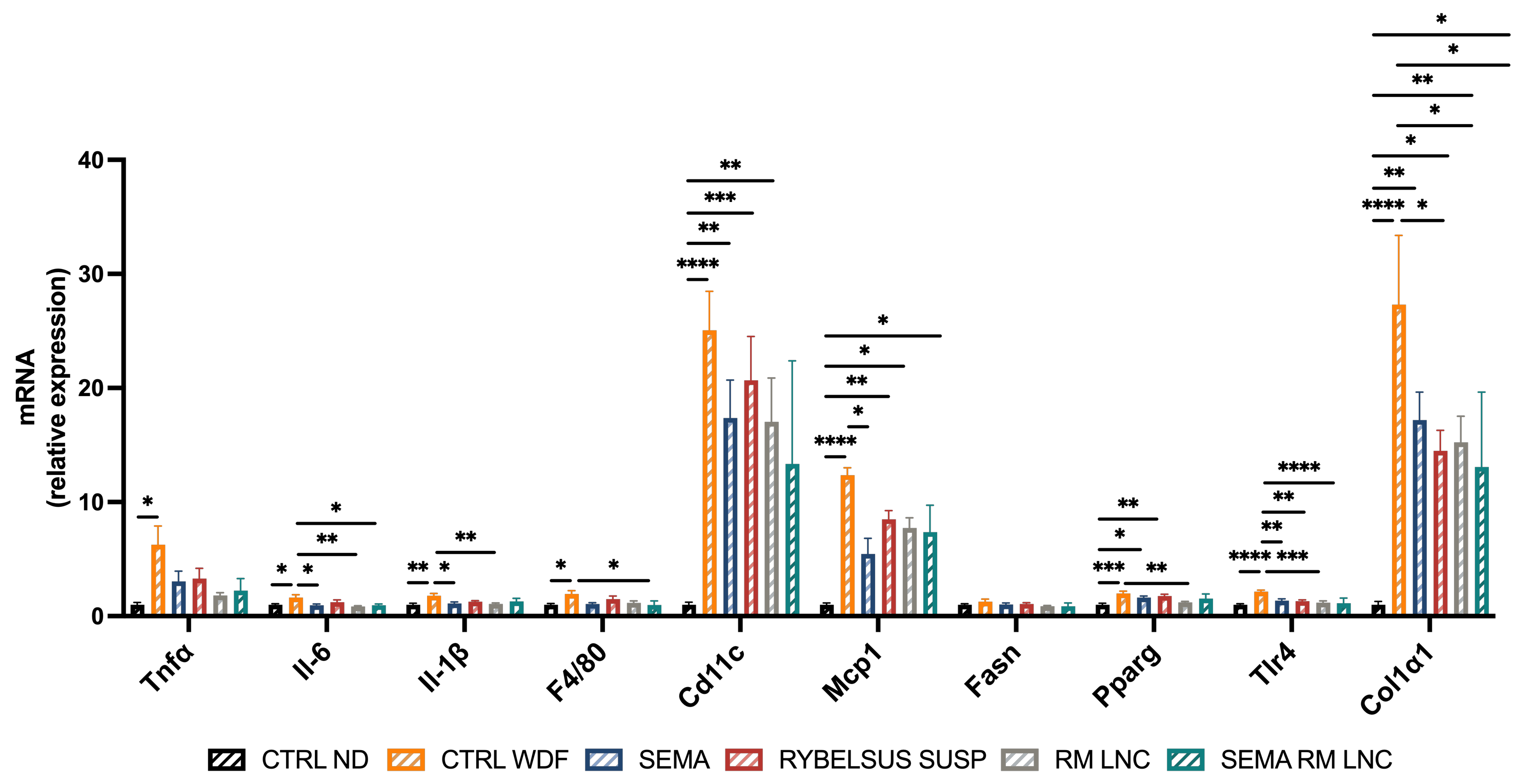


**Figure S5:** SEMA-RM-LNC have an impact on liver inflammation in the WDF model of early NASH. (A) Relative expression mRNA normalized to the CTRL ND group. P values were determined by One-way Anova followed by Tukey’s post hoc test or by Kruskal-Wallis followed by Dunn’s post hoc test (**P*<0.05, ***P<*0.01, ****P<*0.001, *****P<*0.0001).Data represented as mean±SEM (n=9-10).

**Table S1:** Primer Sequences for gene expression analyses by RT-qPCR

|  | Forward primer | Reverse primer |
| --- | --- | --- |
| *Rpl19* | GAAGGTCAAAGGGAATGTGTTC | CCTTGTCTGCCTTCAGCTTGT |
| *Tnfα* | GTGCCTATGTCTCAGCCTCTT | GCTCATACCAGGGTTTGAGCT |
| *Il-6* | CTGCAAGAGACTTCCATCCAGTT | GAAGTAGGGAAGGCCGTGG |
| *Il-1β* | AGTTGACGGACCCCAAAAGA | GGACAGCCCAGGTCAAAGG |
| *F4/80* | GATGATTTCCCGTGTTGTTGGT | ACATCAGTGTTCCAGGAGACACA |
| *Cd11c* | ACGTCAGTACAAGGAGATGTTGGA | ATCCTATTGCAGAATGCTTCTTTACC |
| *Mcp1* | CCACTCACCTGCTGCTACTCAT | CTGCTGGTGATCCTCTTGT |
| *Fasn* | GATCCTGGAACGAGAACACGAT | AGAGACGTGTCACTCCTGGACTT |
| *Pparg* | CTGCTCAAGTATGGTGTCCATGA | TGAGATGAGGACTCCATCTTTATTCA |
| *Tlr4* | ACCTGGCTGGTTTACACGTC | CTGCCAGAGACATTGCAGAA |
| *Col1a1* | GACTGGAAGAGCGGAGAGTACTG | CAGGTCTGGACCTGTCTCCATGTT |
